# Supplementary material for: End Sequence Analysis Toolkit (ESAT) expands the extractable information from single-cell RNA-seq data
Source: Genome Res. 2016 Oct;26(10):1397–410. doi: 10.1101/gr.207902.116 (PMC5052061; doi:10.1101/gr.207902.116)
Supplement: Supplemental Material [file supp_gr.207902.116_Supp_Materials.tar.gz › ESAT-esat_v0.1/doc/html/classbroad_1_1core_1_1primer3_1_1_primer3_exception.html]

ESAT: broad.core.primer3.Primer3Exception Class Reference


ESAT


- Main Page
- Related Pages
- Packages
- Classes
- Files

- Class List
- Class Index
- Class Hierarchy
- Class Members

All Classes Namespaces Files Functions Variables Enumerator Pages

- broad
- core
- primer3
- Primer3Exception

Public Member Functions |
List of all members

broad.core.primer3.Primer3Exception Class Reference

Inheritance diagram for broad.core.primer3.Primer3Exception:

[legend]

Collaboration diagram for broad.core.primer3.Primer3Exception:

[legend]

|  |  |
| --- | --- |
| Public Member Functions | |
|  | Primer3Exception (String message) |
|  | |
|  | Primer3Exception (Throwable cause) |
|  | |

## Detailed Description

Exception thrown when there is a problem generating primers using primer3.

Author
:   Andrew R. Zimmer

## Constructor & Destructor Documentation

|  |  |  |  |  |  |
| --- | --- | --- | --- | --- | --- |
| broad.core.primer3.Primer3Exception.Primer3Exception | ( | String | *message* | ) |  |

Creates a new Primer3Exception with the the given message.

Parameters
:   |  |  |
    | --- | --- |
    | message | An error message describing the nature of the problem. |

|  |  |  |  |  |  |
| --- | --- | --- | --- | --- | --- |
| broad.core.primer3.Primer3Exception.Primer3Exception | ( | Throwable | *cause* | ) |  |

Creates a new Primer3Exception with a Throwable that was the underlying cause of the problem.

Parameters
:   |  |  |
    | --- | --- |
    | cause | the Throwable that was the underlying cause of the problem. |

---

The documentation for this class was generated from the following file:

- java/broad/core/primer3/Primer3Exception.java


---

Generated on Fri Aug 15 2014 15:10:55 for ESAT by  

 1.8.7
